# Multimorbidity patterns and the association with health status of the oldest-old in long-term care facilities in China: a two-step analysis

**DOI:** 10.1186/s12877-023-04507-8

**Published:** 2023-12-13

**Authors:** Hong-Li Chen, Xiao-Hong Yu, Yue-Heng Yin, En-Fang Shan, Ying Xing, Min Min, Ya-Ping Ding, Yang Fei, Xian-Wen Li

**Affiliations:** 1 School of Nursing, Nanjing Medical University, Nanjing, Jiangsu Province China; 2 Landsea Long-Term Care Facility, Nanjing, Jiangsu province China; 3 Xia Man Yun Jian Social Welfare Development Centre, Shanghai, China

**Keywords:** Multimorbidity pattern, Health status, Oldest-old, Younger-old, Long-term care facility

## Abstract

**Background:**

The increasing prevalence of multimorbidity has created a serious global public health problem in aging populations. Certain multimorbidity patterns across different age ranges and their association with health status remain unclear. The main aim of this study is to identify multimorbidity patterns discrepancies and associated health status between younger-old and oldest-old.

**Methods:**

The Ethics Committee of Nanjing Medical University approved the study protocol (No.2019–473). Convenience sampling method was used to recruit older adults aged ≥ 60 years with multimorbidity from July to December 2021 from 38 Landsea long-term care facilities in China. The multimorbidity patterns were analyzed using network analysis and two-step cluster analysis. One-Way ANOVA was utilized to explore their association with health status including body function, activity of daily living, and social participation. A Sankey diagram visualized the flow of health status within different multimorbidity patterns. This study is reported following the STROBE guidelines.

**Results:**

A total of 214 younger-old (60–84 years) and 173 oldest-old (≥ 85 years) were included. Leading coexisting diseases were cardiovascular disease (CD), metabolic and endocrine disease (MED), neurological disease (ND), and orthopedic disease (OD). Cluster 1 (53, 24.8%) of CD-ND (50, 94.3%; 31, 58.8%), cluster 2 (39, 18.2%) of MED-ND-CD (39, 100%; 39, 100%; 37, 94.9%), cluster 3 (37, 17.3%) of OD-CD-MED-ND (37, 100%; 33, 89.2%; 27, 73.0%; 16, 43.2%), and cluster 4 (34, 15.9%) of CD-MED (34, 100%; 34, 100%) were identified in the younger-old. In the oldest-old, the primary multimorbidity patterns were: cluster 1 (33, 19.1%) of CD-respiratory disease-digestive disease-urogenital disease (CD-RD-DSD-UD) (32, 97.0%; 9, 27.3%; 8, 24.2%; 7, 21.2%), cluster 2 (42, 24.3%) of ND-CD-MED (42, 100%; 35, 83.3%; 14, 33.3%), cluster 3 (28, 16.2%) of OD-CD-MED (28, 100%; 25, 89.3%; 18, 64.3%), and cluster 4 (35, 20.2%) of CD-MED (35, 100%; 35, 100%). Younger-old with CD-ND or MED-ND-CD, and oldest-old with ND-CD-MED have worse health status compared with other multimorbidity patterns (e.g., CD-MED and OD-CD-MED).

**Conclusion:**

Discrepancies in common patterns of multimorbidity across age groups suggest that caregivers in long-term care facilities should consider changes in multimorbidity patterns with ageing when developing prevention plans for individualized management. Neurological disease concurrent with other diseases was the major determinant of health status, especially for the oldest-old. Interventions targeting multimorbidity need to be focused, yet generic. It is essential to assess complex needs and health outcomes that arise from different multimorbidity patterns and manage them through an interdisciplinary approach and consider their priorities to gain high-quality primary care for older adults living in long-term care facilities.

**Supplementary Information:**

The online version contains supplementary material available at 10.1186/s12877-023-04507-8.

## Background

With the aging of the global population, more than half of the older adults have been affected by multimorbidity [1]. Moreover, numbers of the oldest-old aged 85 years and over are expected to double by 2035 [2], with multimorbidity the norm in this age group [3]. The term multimorbidity widely refers to the existence of numerous medical conditions in a person and indicates that no single disease holds priority over any co-occurring diseases, which also concerns the patient as the center. Whereas comorbidity mainly interests in an index disease and the possible effects of other disorders [4]. Multimorbidity, therefore, is a more patient-centered concept and takes into account all coexisting diseases being equal importance with interactions. The increasing prevalence of multimorbidity has created a serious public health problem due to its association with functional decline, poorer quality of life, increased risk of premature disability [5, 6]. The impact of multimorbidity on the individual’s health surpasses the impact of the summed effect of single chronic disease. This nonlinear pattern may be further exacerbated by a continuous imbalance between illness and the ability of people with multimorbidity to cope with such a burden, which leads to a vicious cycle of breakdowns in health outcomes [7].

The impact of multimorbidity on health status was not only determined by the number of co-existing diseases [8] but also probably related to the multimorbidity patterns. For example, people with osteoarthritis were associated with a greater risk of hospitalization and death in combination with cardiovascular and musculoskeletal than with musculoskeletal and mental health [9]. Moreover, according to analysis of Swedish aging population, co-occurrence of different neuropsychiatric diseases was major determinant of disability and slow walking speed, whereas isolated cardiovascular multimorbidity impacted only a decline in walking speed [10]. These may be related to diseases that belong to multimorbidity patterns interacting with each other and resulting more severe functional limitations. The relevance between the number of diseases and health outcomes of older adults has been studied in detail. However, it is vital to study the multimorbidity patterns. It can reveal concordant and discordant patterns, hence promote a better understanding of co-occurring diseases and provide an essential information for developing guidelines which can offer coordinated and integrated management and treatment decision support.

Age has been found to be a strong associated factor of the multimorbidity patterns [11, 12]. One study investigating rural community-resident aged 60 years and older revealed a four-cluster multimorbidity structure. However, the research objects of this study mainly comprised the younger-old [13]. Another research on Swedish older people aged 76 years old and above reported five main multimorbidity patterns (e.g., circulatory and cardiopulmonary, visual impairment and anemia, mental and musculoskeletal disorders) [14], while it reported the multimorbidity patterns of all age groups of older adults without the comparison. The comparison of multimorbidity patterns between younger-old and oldest-old with direct evidence is vital for a credible conclusion on change over time, which is because with the continuous accumulation of pathogenic factors, age-related diseases and worse health status are more often seen in the oldest-old, especially for the older adults in long-term care facilities, whose care dependency levels become too complex or costly to be met at home by the available community services.

Previous studies on multimorbidity mainly paid close attention to the impact on the physical function of older adults, which largely hindered the comprehensive understanding of health status including cognition, activity, and social participation [15, 16]. The International Classification of Functioning, Disability, and Health (ICF) framework developed by World Health Orgnization (WHO) is a promising tool to establish the overall health structure of older adults with multimorbidity which could increase our understanding of who is at risk of poor health status [17]. Under the ICF framework, disorder or disease is a condition that develops as a process with the potential to impair three aspects of health status in terms of physical structure, activities and social participation [18, 19].

In light of the complexity of the impact of different multimorbidity patterns on health status and the heterogeneity caused by age-related factors is still limited. The aim of this study was to take a two-step clustering approach to (1) explore the discrepancies of multimorbidity clusters across different age ranges; (2) clarify the effects of different multimorbidity patterns on the health status of the older adults in long-term care facilities in China. We hypothesized that (1) multimorbidity patterns in the oldest-old would be different from those younger-old; (2) diseases would have inequality effects on health status by different multimorbidity patterns. This study could provide valuable information for clinicians and caregivers to predict the prognosis of older adults with multimorbidity and provide evidence support for developing multimorbidity management programs for the healthcare of older adults in long-term care facilities.

## Methods

### Study design and participants

A cross-sectional study was conducted to explore multimorbidity present in older adults and the associated factors and health outcomes. According to the WHO recommendation that individuals older than 60 should be considered older adults in developing countries, subjects included in the study needed to meet both of the following inclusion criteria: (1) aged ≥ 60 years; (2) diagnosed with two or more diseases. Participants were excluded if: (1) they subjective report but without a definite diagnosis of disease; (2) the older adults or their legal guardian refused to participate in this study; (3) they had lived in a long-term care facility for less than 6 months. Cluster analysis sample size requires that the cluster sample size be at least 10 times the number of clustered variables [20]. The number of variables in our study is nine (nine diseases), indicating that a total of 90 older adults would be required in clustering procedure for each age group. In addition, previous studies have shown that older adults with multimorbidity of visual impairment and cognitive impairment were associated with 3 to 6 times greater odds of disability [21]. When OR = 3, Cohen's *f* = 0.30. Effect size conversion was performed by an online effect size conversion tool (https://www.psychometrica.de/effect_size.html) [22]. One-way ANOVA a priori analysis conducted through G-power software showed that 128 older adults were needed in each age group when a = 0.05, power = 0.80, *f* = 0.30, and the number of subgroups was 4 (4 is the median number of subgroups in the clustering scheme) [23].

### Data collection

We used a convenience sampling method and collected data from July to December 2021 based on the Caring Information System of 38 Landsea long-term care facilities across Beijing, Shanghai, Nanjing, Hangzhou, and Suzhou cities in China. Care Information System is a health information collection tool during institutionalization, which comprehensively incorporates demographic data and all information related to the health assessment of each older adult. Also, healthcare workers are required to supplement and update their health information in this system. During the data collection period, we collected the latest data on older adults from the system. The questionnaire survey was completed by health professionals who were trained to conduct the assessment ahead. Respondents were older residents or caregivers if the participants could not answer. To ensure the orderly conduct of the investigation, a standard operating procedure was created to provide uniform training to the investigators. The data collection in this study strictly adhered to the regulations of the Ethics Committee of Nanjing Medical University (No.2019–473) and written informed consent was obtained from all respondents.

### Measurements

The ICF health-related domains, including body function, activity, and participation were served as starting point to understand health status in older adults with multimorbidity. ICF body function was measured by Mini-Mental State Examination (MMSE), Berg Balance Scale (BBS), and Sensory Perception and Communication Scale (SPCS). Barthel Index (BI) was used to measure ICF activity and ICF participation was evaluated by Social Participation Scale (SPS).

#### Multimorbidity

The disease diagnosis certificate was required to provide to health professionals to identify the diseases of older adults. As well, information about disease status was asked by health professionals with questions “Have you been diagnosed with any diseases by the doctor? What medication and treatment are you receiving at present?”. A total of 9 types of diseases were used to measure multimorbidity, including cardiovascular disease, respiratory disease, metabolic and endocrine disease, digestive system disease, orthopedic disease, neurological disease, urogenital disease, hematological disease, and cancer.

#### Cognitive function

The Chinese version of the MMSE was used to measure the cognitive function. The MMSE is a standardized cognitive screening test with a possible score of 0–30. The domains include temporal and spatial orientation, memory, attention and computation ability, recall ability, language skills, and structural copying [24]. Higher the total score, indicate the better cognitive function.

#### Balance

The Chinese version of the BBS with high sensitivity and specificity for predicting fall risk in the Chinese older adults was utilized to test for balance. It evaluates both dynamic and static balance through 14 tasks regarding mobility. Each task is graded on a 5-point ordinal scale that ranges from 0 to 4 for a maximum score of 56. In general, a score of 0 is given when the individual is unable to perform the task, and a score of 4 is given when able to complete the given task independently [25].

#### Sensory perception and communication

The SPCS, developed by the Ministry of Civil Affairs of the People’s Republic of China and defined as the nationally recommended assessment standard, was used to measure individual ability in vision, hearing, consciousness and communication. Visual and hearing are likely to be scored on a scale of 0–4, while consciousness and communication score ranges from 0 to 3, with higher scores in each item indicating lower perception and communication skills [26].

#### Activities of Daily Living (ADL)

The functional limitations were assessed using the Chinese version of the BI for ADL measurement [27]. The BI contains 10 items: bowel control, bladder control, grooming, bathing, toilet use, dressing, feeding, stair climbing, transferring from bed to chair, and mobility. The total ADL score ranged from 0 to 100. A higher ADL score reflected a higher level of independence.

#### Social participation

The SPS was also issued by the Ministry of Civil Affairs of the People’s Republic of China [26]. The scale includes five dimensions of family life, work, temporal/spatial orientation, personal orientation, and social interaction, which is a nationally recommended assessment tool. A score from 0 to 4 is given for each item, with higher scores indicating worse social engagement.

### Statistical analysis

Data were analyzed by using the IBM SPSS Statistics Version 27. Regression was used to impute the missing values for age and education. The following descriptive statistics were calculated for the analysis of demographic variables and health status: mean, standard deviation, number (n), and percentage (%). Chi-square test and T-test were performed to test differences in these variables across age groups (alpha = 0.05). Network analysis of multimorbidity was performed using Gephi (version 0.9.3). A two-step cluster analysis method with noise handling was chosen to detect latent relationships within and between multimorbidity clusters. The two‑step cluster analysis, as an exploratory tool, is mainly used to reveal natural groupings (or clusters) within a dataset that would otherwise not be apparent. For multimorbidity where groupings are not known a priori, two-step clustering will identify clustered disease patterns first through preclustering and followed by hierarchical clustering. Because it is explorative, it does not make any assumptions about the results. In addition, two-step clustering is an intelligent clustering method that automatically selects the optimal number of clusters based on the value of ratio of distance measure and Bayesian information criteria (*BIC*) between adjacent clusters and provides silhouette measure of cohesion and separation to test the overall goodness-of-fit of the model. The silhouette measure values between 0.2 and 0.5 indicates fair and greater than 0.5 indicates good solution quality [28]. Then, we applied multinomial logistical regression to explore the association between the independent variables of demographic characteristics and the different clusters of multimorbidity. One-Way Analysis of Variance (ANOVA) with post hoc Least-Significant Difference (LSD) tests was employed for the correlation between multimorbidity patterns and health status. The level of significance for rejecting the null hypothesis was alpha = 0.05. A Sankey diagram was performed to visualize the flow of health status in different multimorbidity patterns.

## Results

### Demographic characteristics, disease conditions, and health status of the participants

The final sample consisted of 387 older adults of whom 214 were younger-old (60–84 years old) and 173 were oldest-old (≥ 85 years old). They were between 61 and 101 years of age (82.55 ± 7.44 years). With a balanced gender distribution, nearly half of both younger-old and oldest-old were male (120, 56.1% and 82, 47.4%, respectively). Significant differences in mean scores for sensory perception and communication (*t* = -2.066, *p* = 0.040) were observed between these two groups (Table 1). The Cronbach’s alpha of all the scales in this study was from 0.678 to 0.978, indicating acceptable internal consistency.

**Table 1:** Descriptive of included older adults [n (%)/M ± SD]

Characteristics	All (*n* = 387)	Age Group		*p*-value
		60–84 (***n*** = 214)	≥ 85 (***n*** = 173)	
**Gender**				
Male	202 (52.2)	120 (56.1)	82 (47.4)	0.089
Female	185 (47.8)	94 (43.9)	91 (52.6)	
**Education**				
≤ Primary	113 (29.2)	64 (29.9)	49 (28.3)	0.299
Middle	88 (22.7)	54 (25.2)	34 (19.7)	
≥ Bachelor	186 (48.1)	96 (44.9)	90 (52.0)	
**Number of multimorbidity**				
2	131 (33.9)	65 (30.4)	66 (38.2)	0.381
3	95 (24.5)	53 (24.8)	42 (24.3)	
4	74 (19.1)	43 (20.1)	31 (17.9)	
≥ 5	87 (22.5)	53 (24.8)	34 (19.7)	
**Type of disease**				
Cardiovascular disease	328 (84.8)	185 (86.4)	143 (82.7)	0.302
Respiratory disease	74 (19.1)	38 (17.8)	36 (20.8)	0.448
Metabolic and Endocrine disease	216 (55.8)	129 (60.3)	87 (50.3)	**0.049**
Digestive system disease	50 (12.9)	24 (11.2)	26 (15.0)	0.266
Orthopedic disease	108 (27.9)	51 (23.8)	57 (32.9)	**0.047**
Neurological disease	173 (44.7)	108 (50.5)	65 (37.6)	**0.011**
Urogenital disease	86 (22.2)	51 (23.8)	35 (20.2)	0.397
Hematological disease	17 (4.4)	12 (5.6)	5 (2.9)	0.195
Cancer	15 (3.9)	12 (5.6)	3 (1.7)	0.050
MMSE	21.81 ± 8.84	21.94 ± 8.81	21.65 ± 8.90	0.743
BBS	28.18 ± 19.05	29.48 ± 19.28	26.56 ± 18.68	0.134
SPCS	2.42 ± 2.27	2.21 ± 2.19	2.69 ± 2.35	**0.040**
BI	65.00 ± 28.77	66.50 ± 28.83	63.15 ± 28.67	0.256
SPS	7.28 ± 5.80	7.14 ± 5.90	7.46 ± 5.68	0.594

*Abbreviations*: *M* Mean, *SD* Standard deviation, *MMSE* Mini-mental state examination, *BBS* Berg balance scale, *SPCS* Sensory perception and communication Scale, *BI* Barthel index, *SPS* Social participation scale

*p* < 0.05 highlighted in bold font

Leading existing diseases reported were cardiovascular disease (328, 84.8%), metabolic and endocrine disease (216, 55.8%), neurological disease (173, 44.7%), and orthopedic disease (108, 27.9%). The prevalence of most diseases was similar in the two age groups. However, the prevalence of orthopedic disease was identified as 23.8% (52) in the younger-old group to 32.9% (57) in the oldest-old group, showing a significantly increasing sequence (χ^2^ = 3.952, *p* = 0.047). In contrast, the prevalence rates of metabolic and endocrine disease (χ^2^ = 3.872, *p* = 0.049), and neurological disease (χ^2^ = 6.435, *p* = 0.011) showed a significantly decrease from younger-old (129, 60.3% and 108, 50.5%, respectively) to oldest-old groups (87, 50.3% and 65, 37.6%, respectively) (Table 1).

**Fig. 1:**
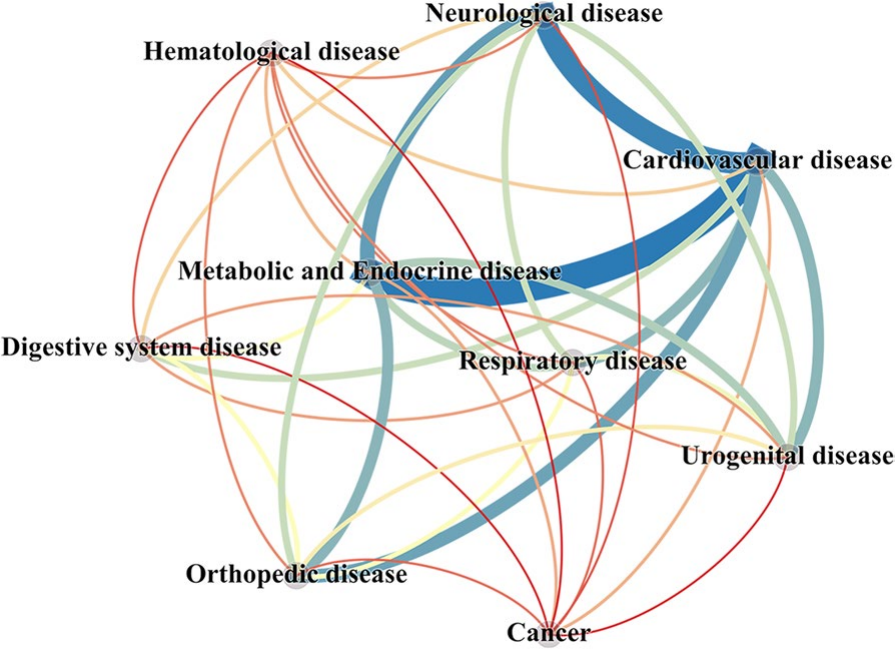
Network analyses of multimorbidity in all the participants

The network analysis of 9 diseases is shown in Fig. 1, with 9 diseases constituting 36 network edges. An edge of weight = 1 between two nodes represents a single subject with both multimorbidity. The thicker the edge, the greater number of older adults with the same of multimorbidity patterns. The network was dominated by cardiovascular disease, metabolic and endocrine disease, neurological disease, and orthopedic disease, with frequent co-occurrence linkages between these diseases.

### Clusters of multimorbidity in younger-old and oldest-old groups

**Fig. 2:**
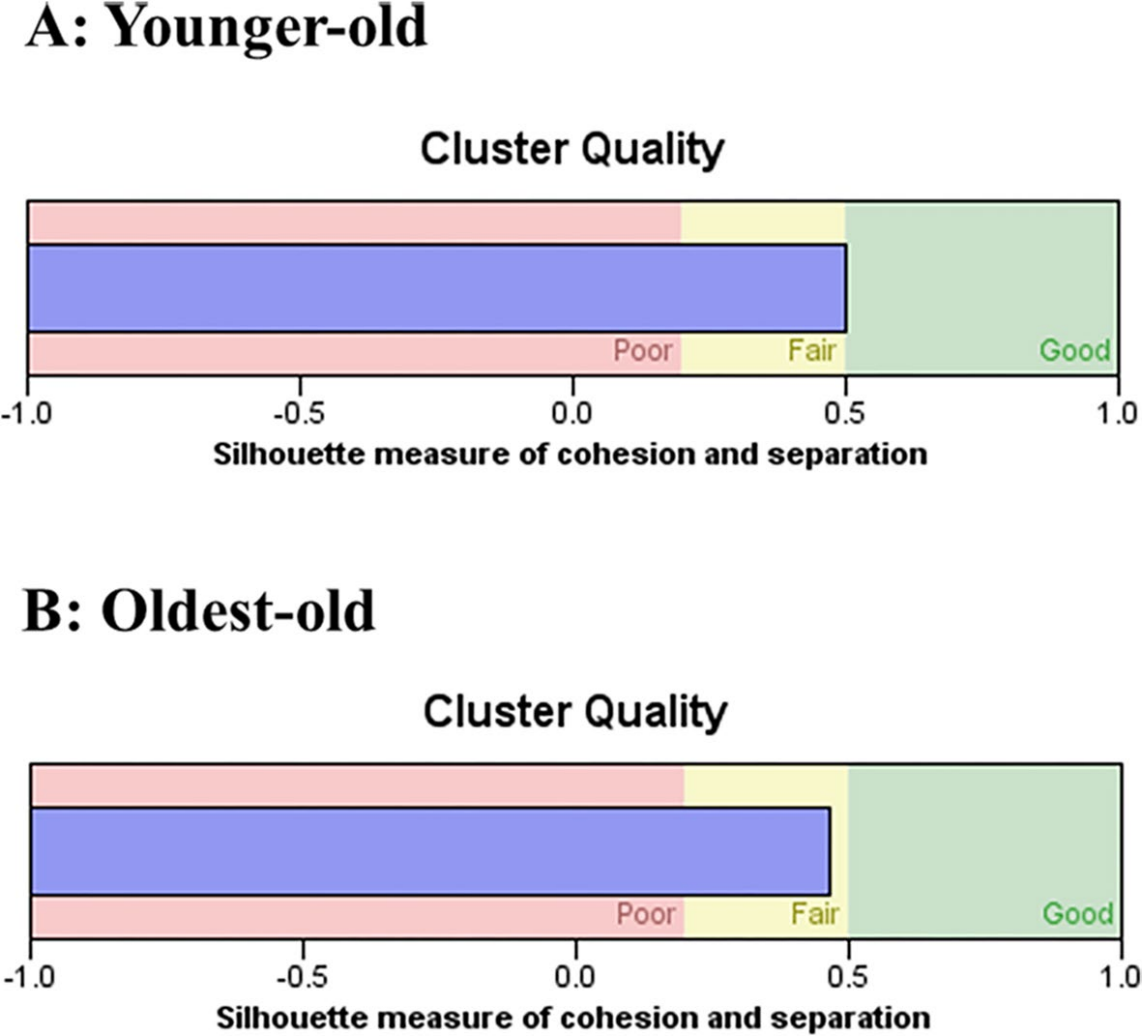
The two-step cluster analysis of multimorbidity older adults

Two-step cluster analysis showed that the four-cluster model of multimorbidity was the optimal clustering solution in younger-old and oldest-old groups according to the ratio of distance measure and *BIC* value of different clusters. In the two groups, the four clusters solution gave the highest value for the ratio of distance measure (younger-old: 1.921, oldest-old: 2.538) and the lowest *BIC* value (younger-old: 225.456, oldest-old: 299.518) (see Additional file 1). The silhouette measure of cohesion and separation were 0.5, respectively, within the fair range (Fig. 2).

**Fig. 3:**
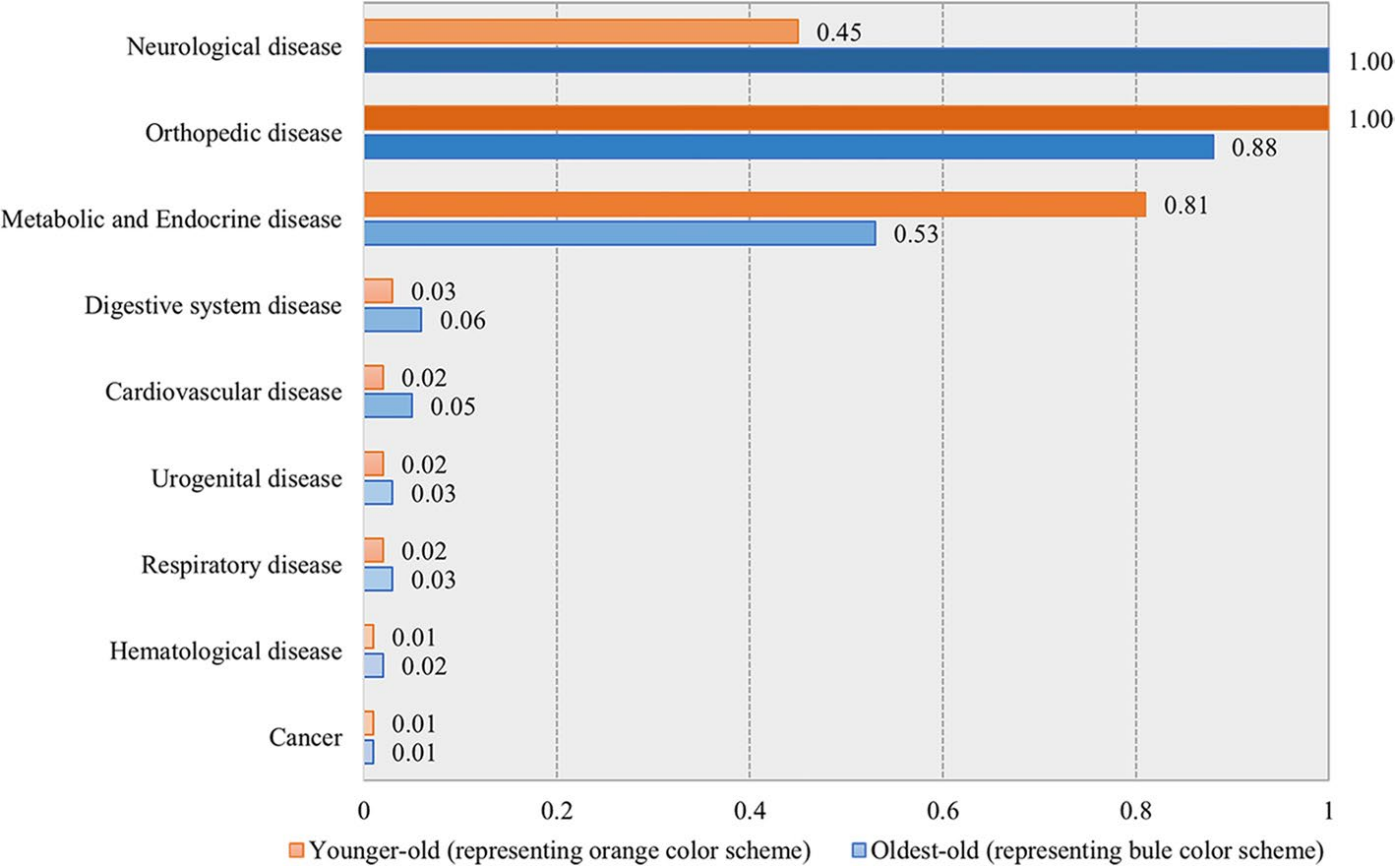
The predictor importance of the diseases in making the clusters

As for the importance of the diseases in the clustering procedure, higher values associated with a disease indicate a greater discriminative capacity for the indicator. The metabolic and endocrine disease had a value of 0.81 and 0.53 in the younger-old and oldest-old groups, respectively, indicating that it was important in the clustering procedure of the two groups. In the younger-old group, orthopedic disease (1.00) had the highest discriminating power to form cluster division, while in the oldest-old group, the value of orthopedic disease was 0.88. In contrast, the neurological disease had the highest value of 1.00 in the oldest-old group, whereas the value was 0.45 in the younger-old group. Cardiovascular disease (0.02 and 0.05, respectively) played a less prominent role in the clustering procedure (Fig. 3).

**Fig. 4:**
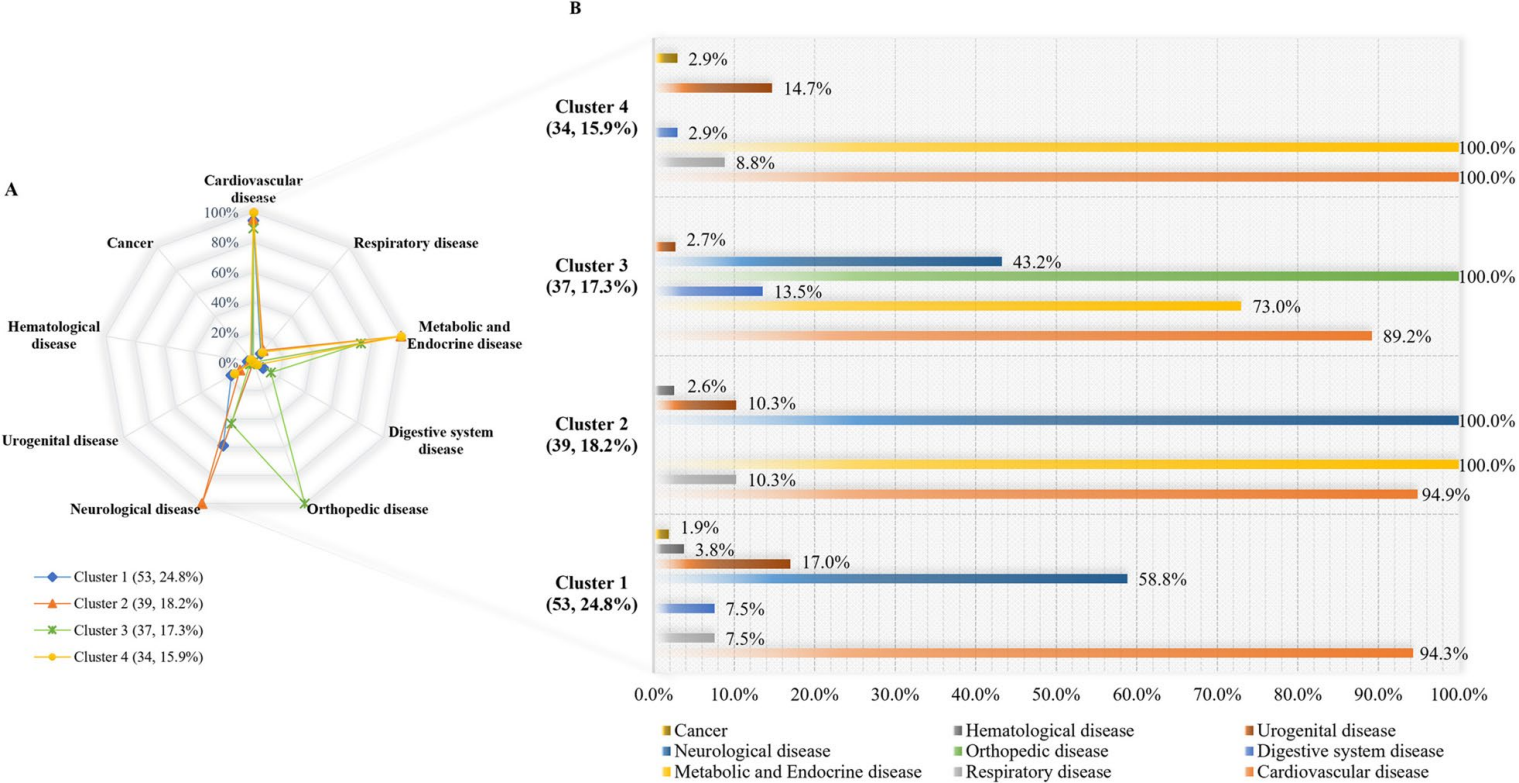
Multimorbidity distribution of each cluster in younger-old

As shown in Fig. 4, there were 163 cases in the younger-old group included in the clustering procedure, while there were 51 cases were excluded due to the use of noise handling. Cluster 1 achieved the largest sample (53, 24.8%), with the most prominent features of this cluster including the presence of cardiovascular diseases (50, 94.3%) and neurological disease (31, 58.8%) (CD-ND). Cluster 2 consisted of 39 (18.2%) younger-old who had a high rate of metabolic and endocrine disease (39, 100%), neurological disease (39, 100.0%), and cardiovascular disease (37, 94.9%) (MED-ND-CD). Cluster 3 (37, 17.3%) was characterized by the younger-old with orthopedic disease (37, 100.0%), cardiovascular disease (33, 89.2%), metabolic and endocrine disease (27, 73.0%), and neurological disease (16, 43.2%) (OD-CD-MED-ND). Cluster 4 (34, 15.9%) was presented with cardiovascular disease (34, 100.0%), metabolic and endocrine disease (34, 100.0%) (CD-MED).

**Fig. 5:**
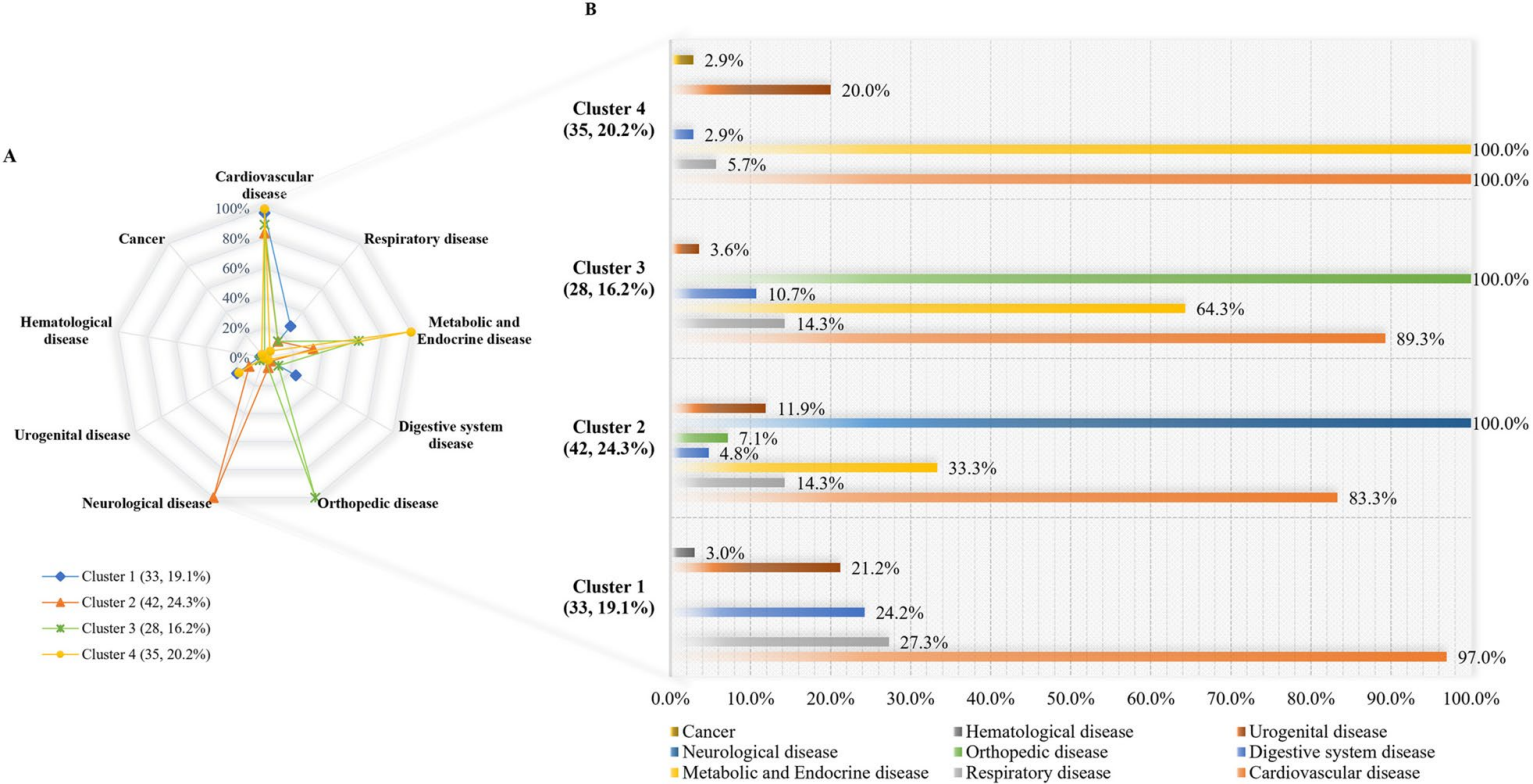
Multimorbidity distribution of each cluster in oldest-old

The detailed distribution of the diseases in the oldest-old is given in Fig. 5. The sample of four clusters was 33 (19.1%), 42 (24.3%), 28 (16.2%), and 35 (20.2%), respectively. Thirty-five cases were excluded due to the use of noise handling. The most distinctive diseases in cluster 1 were cardiovascular disease (32, 97.0%), followed by respiratory disease (9, 27.3%), digestive system disease (8, 24.2%), and urogenital disease (7, 21.2%) (CD-RD-DSD-UD). The multimorbidity pattern of the oldest-old in cluster 4 was similar to that of the younger-old in cluster 4, characterized by the high representation of cardiovascular disease (35, 100.0%) and metabolic and endocrine disease (35, 100.0%) (CD-MED). As well, the oldest-old in cluster 3 had a similar multimorbidity pattern to cluster 3 in the younger-old group (OD-CD-MED-ND), while with different proportions of cardiovascular disease (25, 89.3%), metabolic and endocrine disease (18, 64.3%), and without neurological disease (OD-CD-MED). The most prominent features of cluster 2 were neurological disease (42, 100%), followed by cardiovascular disease (35, 83.3%), and metabolic and endocrine disease (14, 33.3%) (ND-CD-MED).

### Association between clusters of multimorbidity and the demographic characteristics in different age groups

The sociodemographic characteristics across the clusters in older adults are provided in Table 2. In the multinominal regression analysis, males had a lower association with the OD-CD-MED-ND (OR: 0.137, 95% CI: 0.047–0.400) and OD-CD-MED (OR: 0.255, 95% CI: 0.086–0.759) clusters than females when compared with the CD-MED cluster, which both emerged in the younger-old and oldest-old groups. As well, oldest-old with middle education degree had a lower association with multimorbidity of OD-CD-MED (OR: 0.205, 95% CI: 0.046–0.919) than those with bachelor or above degrees when compared with the CD-MED. There was no significant difference between other clusters in sociodemographic variables.

**Table 2:** Demographic variables of older adults and the association across the clusters

**Age group**	**Cluster**		**Gender**		**Education**		
			**Male**	**Female**	** ≤ Primary**	**Middle**	** ≥ Bachelor**
Younger-old (n = 163)	CD-ND	n (%)	31 (58.5)	22 (41.5)	14 (26.4)	17 (32.1)	22 (41.5)
		OR (95% CI)	0.691 (0.279–1.712)	Reference	1.421 (0.487–4.146)	1.698 (0.594–4.850)	Reference
	MED-ND-CD	n (%)	22(56.4)	17(43.6)	15 (38.5)	8 (20.5)	16 (41.0)
		OR (95% CI)	0.622 (0.237–1.631)	Reference	2.088 (0.699–6.236)	1.091 (0.331–3.600)	Reference
	OD-CD-MED-ND	n (%)	8 (21.6)	29 (78.4)	13 (35.1)	13 (35.1)	11 (29.7)
		OR (95% CI)	0.137(0.047–0.400) *	Reference	2.548 (0.760–8.546)	2.350 (0.700–7.892)	Reference
	CD-MED1	n (%)	23 (67.6)	11 (32.4)	8 (23.5)	8 (23.5)	18 (52.9)
		OR (95% CI)	1	1	1	1	1
Oldest-old (n = 138)	CD-RD-DSD-UD	n (%)	15 (45.5)	18 (54.5)	14 (42.4)	5 (18.5)	14 (42.4)
		OR (95% CI)	0.567 (0.215–1.499)	Reference	1.038 (0.350–3.079)	0.464 (0.124–1.734)	Reference
	ND-CD-MED	n (%)	20 (47.6)	22 (52.4)	10 (23.8)	9 (21.4)	23 (54.8)
		OR (95% CI)	0.585 (0.233–1.468)	Reference	0.452 (0.152–1.343)	0.508 (0.163–1.581)	Reference
	OD-CD-MED	n (%)	8 (28.6)	20 (71.4)	6 (21.4)	3 (10.7)	19 (67.9)
		OR (95% CI)	0.255 (0.086–0.759) *	Reference	0.310 (0.090–1.071)	0.205 (0.046–0.919) *	Reference
	CD-MED2	n (%)	21 (60.0)	14 (18.9)	12 (34.3)	10 (28.6)	13 (37.1)
		OR (95% CI)	1	1	1	1	1

*Abbreviations:* *CD* Cardiovascular disease, *ND* Neurological disease, *MED* Metabolic and endocrine disease, *OD* Orthopedic disease, *RD* Respiratory disease, *DSD* Digestive system disease, *UD* Urogenital disease, *OR* Odds ratio

^*^p < 0.05 highlighted in bold font

### Health status of different multimorbidity clusters in different age groups

**Fig. 6:**
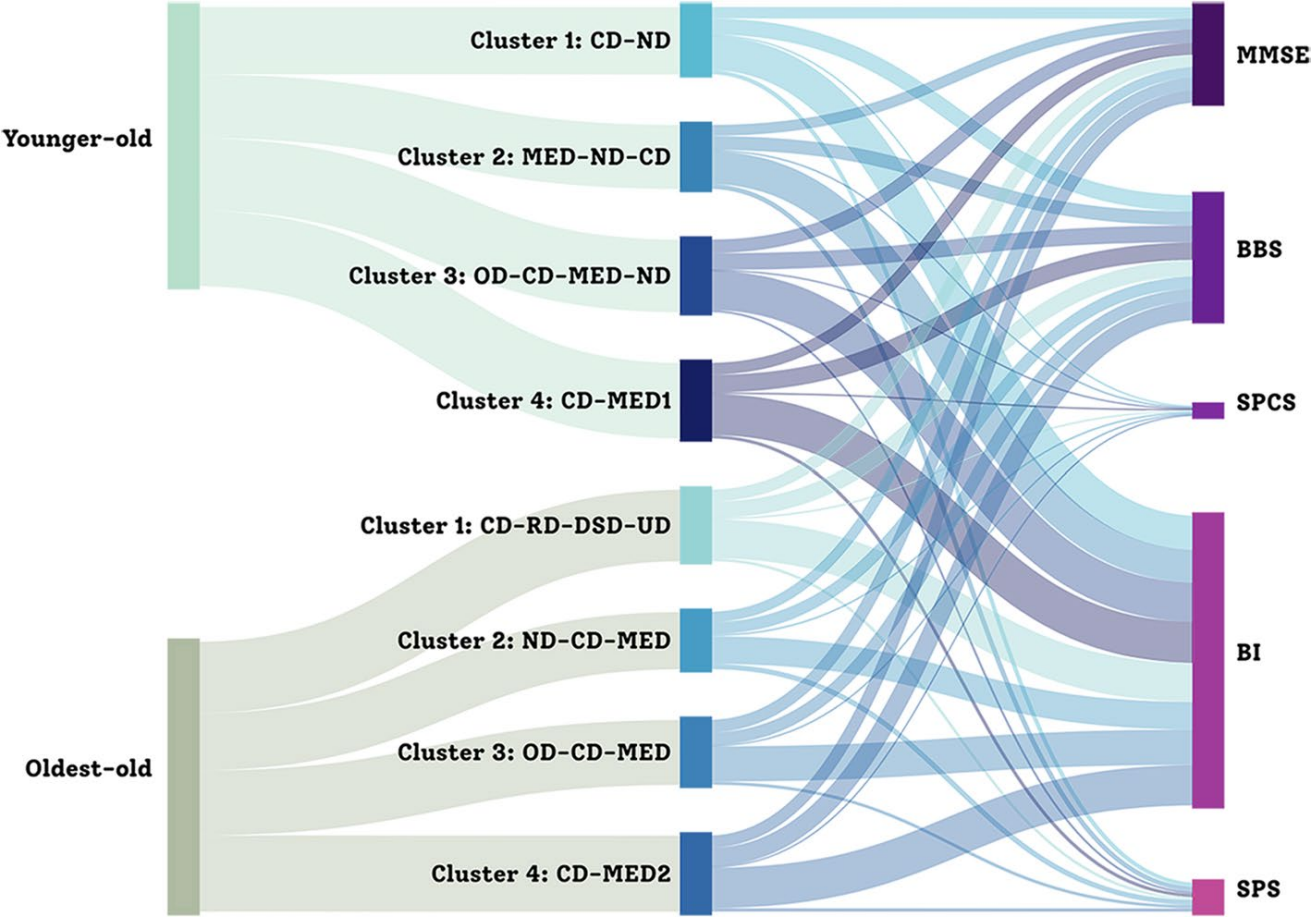
Health status of older adults with different multimorbidity clusters displayed with Sankey Flow Diagrams. CD, Cardiovascular disease; ND, Neurological disease; MED, Metabolic and Endocrine disease; OD, Orthopedic disease; RD, Respiratory disease; DSD, Digestive system disease; UD, Urogenital disease; MMSE, Mini-Mental State Examination; BBS, Berg Balance Scale; SPCS, Sensory Perception and Communication Scale; BI, Barthel Index; SPS, Social Participation Scale

Figure 6 visualizes the health status of older adults with different multimorbidity patterns using Sankey Flow Diagrams. As revealed in Table 3, significant differences were only found in the ADL (*F* = 2.866, *p* = 0.038) and social participation (*F* = 5.135, *p* = 0.002) among four clusters in the younger-old group. Younger-old in the CD-MED cluster were likely to be healthier, especially in these two dimensions than those in the CD-ND and MED-ND-CD clusters. In addition, younger-old in the OD-CD-MED-ND cluster were likely to be less cognitive impairment, less sensory perception and communication deterioration, and better social participation ability than those in the CD-ND and MED-ND-CD clusters which were characterized by a high rate of neurological disease. In the oldest-old, significant differences were found in the balance (*F* = 3.884, *p* = 0.011), sensory perception and communication (*F* = 3.040, *p* = 0.031), ADL (*F* = 6.554, *p* < 0.001) and social participation (*F* = 5.903, *p* = 0.001) among four clusters. Oldest-old with ND-CD-MED had poorer health outcomes than those with other multimorbidity patterns.

**Table 3:** Comparison of health status between clusters

Age group	Cluster	MMSE		BBS		SPCS		BI		SPS	
		**M ± SD**	***F*/*p*-value**	**M ± SD**	***F*/*p*-value**	**M ± SD**	***F*/*p*-value**	**M ± SD**	***F*/*p*-value**	**M ± SD**	***F*/*p*-value**
Younger-old (*n* = 163)	CD-ND	21.30 ± 9.71	2.414/0.069	30.42 ± 19.49	0.958/0.414	2.36 ± 2.30	2.503/0.061	63.77 ± 30.09	2.866/0.038	7.81 ± 6.67	5.135/0.002
	MED-ND-CD	20.49 ± 9.44		26.10 ± 20.63		2.56 ± 2.64		61.41 ± 31.87		8.90 ± 6.03	
	OD-CD-MED-ND	25.43 ± 5.96^ab^		31.46 ± 18.55		1.32 ± 1.49^ab^		73.38 ± 23.66		4.14 ± 4.05^ab^	
	CD-MED1	22.35 ± 8.48		33.32 ± 17.12		2.32 ± 1.95		77.06 ± 20.82^ab^		6.15 ± 5.19^b^	
Oldest-old (*n* = 138)	CD-RD-DSD-UD	22.73 ± 7.26	1.919/0.130	30.79 ± 17.28	3.884/0.011	2.18 ± 1.98	3.040/0.031	72.73 ± 24.43	6.554/ < 0.001	5.30 ± 4.71	5.903/0.001
	ND-CD-MED	19.48 ± 10.73		22.88 ± 19.06		3.36 ± 2.48^a^		51.67 ± 32.32^a^		9.76 ± 6.32^a^	
	OD-CD-MED	23.61 ± 7.30^b^		23.93 ± 18.72		2.04 ± 1.97^b^		65.89 ± 24.84^b^		5.86 ± 4.45^b^	
	CD-MED2	23.09 ± 6.50		35.03 ± 14.26^bc^		2.31 ± 1.91^b^		75.71 ± 19.41^b^		6.09 ± 4.74^b^	

*Abbreviations*: *M* Mean, *SD* Standard deviation, *MMSE* Mini-mental state examination, *BBS* Berg balance Scale, *SPCS* Sensory perception and communication Scale, *BI* Barthel index, *SPS* Social participation Scale, *CD* Cardiovascular disease, *ND* Neurological disease, *MED* Metabolic and Endocrine disease, *OD* Orthopedic disease, *RD* Respiratory disease, *DSD* Digestive system disease, *UD* Urogenital disease

^a^ Significantly (*p* < 0.05) different from the cluster 1, ^b^ Significantly (*p* < 0.05) different from the cluster 2, ^c^ Significantly (*p* < 0.05) different from the cluster 3

## Discussion

This study explored the multimorbidity patterns across different age groups and their associations with health status. We found the network of multimorbidity for older adults was dominated by cardiovascular disease, metabolic and endocrine disease, neurological disease, and orthopedic disease coexisting frequently. Four clusters were identified in either age group, with some similarities and discrepancies compared with each other (Younger-old: CD-ND, MED-ND-CD, OD-CD-MED-ND, CD-MED. Oldest-old: CD-RD-DSD-UD, ND-CD-MED, OD-CD-MED, CD-MED). As for the demographic factors of different multimorbidity patterns, the female showed a higher association with OD-CD-MED-ND in the younger-old group or OD-CD-MED in the oldest-old group when compared with the CD-MED. Additionally, neurological disease concurrent with other diseases were major determinants of health status (e.g., cognitive function, balance, activities of daily life) in older adults, which was particularly obvious in the oldest-old with ND-CD-MED. The ability to balance was also found to be more severely impaired in the OD-CD-MED cluster than in the CD-MED cluster due to the influence of orthopedic disease. Interestingly, we also found that the prevalence rate of cardiovascular disease was generally high among any clusters within both two groups of older adults. However, neurological disease and metabolic and endocrine disease were more commonly seen in the younger-old when compared with the oldest-old, while the orthopedic disease was opposite.

Cardiovascular disease, metabolic and endocrine disease, neurological disease, and orthopedic disease were dominant in the network analysis as a result of their high prevalence rate, and therefore frequent co-occurrence. As for the details, there were differences in multimorbidity patterns in different age groups, and at the same time, there were similarities. CD-MED cluster was commonly seen in the younger-old and oldest-old groups. Other reviews showed cardiometabolic disease was one of the prominent groups [29, 30]. One study provided evidence of metabolic disorders playing an active role in promoting the progression of cardiovascular disease [31]. Several probable mechanisms can be used to illustrate this cluster. Reactive oxygen species and lipid accumulation in metabolic disorders may lead to changes in hemodynamic load, myocardial metabolism, and microvascular dysfunction, which can cause cardiovascular complications [32, 33]. Additionally, the proportions of the diseases in similar multimorbidity patterns between the two groups were various. Comparing MED-ND-CD in the younger-old with ND-CD-MED in the oldest-old, metabolic and endocrine disease played the most prominent role in the two cluster divisions. The result was in line with our finding that metabolic and endocrine disease increase may slow in the oldest-old group. Some studies have demonstrated that metabolic and endocrine disease and cardiovascular disease were related to increased risk of neurological disease such as dementia [34, 35]. Muddapu et al. proposed that any deterioration in the metabolic function may trigger a chain of events (e.g., excess reactive oxygen species, increased inflammation) that precipitate various manifestations of neurodegenerative pathology [36]. Meanwhile, cardiovascular diseases (e.g., hypertension, heart disorders, and dyslipidaemia) are associated with neurological disorders (e.g., dementia and stroke). Several linking features such as hypoxia, amyloid-beta, and oxidative stress have been proposed as a connecting point between them [37]. Research also demonstrated that when the autonomic nervous system is disrupted, it can lead to a variety of cardiovascular issues [38]. These also explain the formation of CD-ND cluster in younger-old. Regarding the multimorbidity patterns containing the orthopedic disease (e.g., osteoarthritis and fractures), OD-CD-MED-ND in the younger-old group and OD-CD-MED in the oldest-old group were presented. This finding was consistent with a UK Clinical Practice Research Datalink study revealing that in individuals with osteoarthritis more common concomitant diseases were cardiovascular disease, mental disease, and metabolic disease [9]. To interpret this pattern, some researchers proposed that lack of exercise, obesity, and diabetes mellitus might have a synergistic adverse effect on the relationship between orthopedic disease (e.g., knee and hip osteoarthritis, osteoporosis) and cardiovascular disease. While there is a need to better understand the potential pathways linking pathophysiological mechanisms between them [39–42]. Meanwhile, Kuusalo et al. also reported that they could not posit a biological rationale for the association of diabetes and cardiovascular disease with prevalent or incident keen osteoarthritis [43]. Kelly et al. found that deficits in the brain can cause deficits in bone and bone itself can affect cognitive function, which may be due to a complex mixture of neuronal, psychological, lifestyle factors, and so on [44]. The mechanisms of coexisting above four types of disease are complex and remain unclear. Finally, the oldest-old may also suffer from a more complex multimorbidity pattern, co-occurring CD-RD-DSD-UD. With aging, there are unavoidable structural, physiological, and immunological changes in these systems and the resultant progressive decline in function and possible poor outcomes. Thus, older adults with multimorbidity (especially co-existing cardiovascular disease, metabolic and endocrine disease, neurological disease, and orthopedic disease) are common. It is vital to deliver health and social care with a focus on integrated interdisciplinary care, including medicine management. Although our results cannot reveal the causality among these diseases. Previous studies we used to interpret could provide several clues, which inform special attention should be paid to satisfying the need to target the appropriate older adults and address their priorities.

Regarding the demographic characteristics associated with various multimorbidity patterns, the female showed a higher association with OD-CD-MED-ND in the younger-old group or OD-CD-MED in the oldest-old group when compared with the CD-MED. Previous studies revealed that the prevalence rate of neurological disease (e.g., dementia) was greater in females (25.16%) than males (18.54%), with dementia rates diverging after age 85 and Alzheimer’s disease (AD) rates diverging around 80. To draw support for the explanation, one reason may be female’s survival longer ages than male’s [45]. Females are also more prone to orthopedic disease such as osteoporosis, fragility fractures, and osteoarthritis, which can be attributed to estrogen deficiency after entering menopause [46]. Considering gender differences in multimorbidity patterns is crucial for diagnosis and treatment. Recognition of these differences can benefit a higher index of suspicion for certain diagnoses. Furthermore, contrary to previous findings which suggested that higher education was associated with a lower risk of knee osteoarthritis [47, 48], our results revealed that middle education level (vs bachelor and above) was associated with the lower prevalence of multimorbidity pattern of OD-CD-MED, when compared to CD-MED in the oldest-old. A cohort study also suggested the inverse association between education and keen/hip osteoarthritis surgery, which were potentially confounded by unobserved familial factors. The high proportion (nearly 50%) of older adults (especially the oldest-old) with bachelor and above education level we sampled may be one explanation. This result must be interpreted with caution. Additionally, in either age group, the potential impact on the overall health status of older adults deepens considerably when they were affected by neurological disease. Compared with CD-ND and MED-ND-CD patterns which were characterized by a high rate of neurological disease, younger-old in the CD-MED pattern were likely to be healthier, especially in the dimensions of ADL and social participation. As well, the results were similar in younger-old with OD-CD-MED-ND who were with a low proportion of neurological disease, except for balance and ADL which are known to be influenced significantly by orthopedic disease. The impact of the neurological disease was similar in the oldest-old group, demonstrating that the ND-CD-MED cluster grouped older adults with worse health status in physical structure (containing cognitive function, sensory perception and communication function, balance), ADL, and social participation compared with any other clusters or single cluster. The ability to balance was also found to be more severely impaired with a significant difference in the OD-CD-MED cluster than in the CD-MED cluster since the influence of orthopedic disease. Empirical evidence has revealed that changes in the brain with cerebral atrophy, neuronal loss, and decrease in several neurotransmitters that accompany the neurological disease are important for effective function in health status including cognition, sensory, balance, ADL, and leisure activities [49, 50]. As we all know, the most common manifestation of neurological disease especially dementia is cognitive degeneration. Due to the long-time progression of disease with aging, it will be more severe, and older adults may require more help with basic activities of daily living (e.g., bathing, eating, and dressing). Several linking mechanisms co-existing with cardiovascular disease, metabolic and endocrine disease may also worsen the symptoms or accelerate the progression rate [51]. Regarding impaired balance, it is also common at diagnosis and becomes more prominent with some neurological diseases (e.g., Parkinson’s disease and epilepsy) progression [52, 53]. In terms of sensory and perception, previous research demonstrated that sensory visual impairments are commonly found in AD due to cortical disturbance. The progressive loss of dopaminergic cells in the retina of the visual system can cause visuoperceptual deficits in older adults with Parkinson’s disease. Physical function impairment in most of neurological disease combined with the composite effect of other diseases may result in impaired ability or less participation in activities [54]. Our findings and the above interpretation imply that more detailed disease names should be clear in future research to identify their more delicate relationships.

Some interesting findings also appeared in the study. Cardiovascular disease played a less prominent role in the clustering on account of the prevalence rate of this disease being generally high among any clusters within both two groups of older adults. Similar to America, the prevalence of cardiovascular disease was over 75% from ages 60–79 years and nearly 90% in older adults above 80 years [55], indicating a considerable high prevalence among all stages of older adults. Age is an independent risk factor for cardiovascular disease, resulting in an increased risk in older adults [56]. The other three diseases had the highest discriminating power to form cluster division and the prevalence of these diseases showed significant differences between the younger-old and oldest-old groups. Neurological disease and metabolic and endocrine disease were more commonly seen in the younger-old compared with the oldest-old, whereas orthopedic disease was opposite. Numerous studies revealed that the prevalence of neurological diseases such as AD and Parkinson’s disease increases substantially with age under the age of 80 or 85 years, after which the increase may slow in the oldest age group [57–59]. The prevalence of AD increases slowed after 80 years, which could be owing to the high prevalence in the oldest ages [60]. A possible interpretation in a previous study for the decrease in the prevalence of Parkinson’s disease among the oldest-old is that it is caused by under-ascertainment of disease among them, since patients are detected through medical records only [58]. The same was observed with metabolic and endocrine disease reporting in a study of the Chinese aging population [61]. For instance, in older adults with diabetes, one study proposed that long-lived humans may have some advantage in glucose handling. Human centenarians had better insulin sensitivity than younger controls who were over 75 years old [62]. The prevalence of orthopedic disease containing fractures and osteoarthritis increases with age which could be attributed to age-related changes in bone and soft tissue being more common. A systematic analysis also revealed that the rates increased steadily through to the oldest age group [63]. The prevalence of these diseases in different age groups varies in different literatures. Due to the limitation of our cross-sectional design, we cannot assert the prevalence of the above diseases is definitely accurate, and the potential explanations, such as healthy people living longer, or some others should be viewed with caution.

For the implications for research, healthcare models are currently focused on measures predominantly managing diseases applied to a single disease and may not be appropriate for multimorbidity patterns. Therefore, updated standardized guidelines for research on prioritized and coordinated interventions for people with multimorbidity are needed, especially for vulnerable older adults [64], which are still lacking. Based on our findings, health caregivers should identify which multimorbidity patterns are more prevalent in older adults (e.g., CD-MED, ND-CD-MED, CD-ND, OD-CD-MED-ND), why certain diseases tend to cluster together, and what differences in heterogeneous patterns may exist in the younger-old and oldest-old. The mechanisms underlying the development of multimorbidity patterns are complex. Although there are many mechanisms such as ageing, inflammation, and oxidative stress may share the same pathways to the development of various diseases. Research on mechanisms underpinning the development of multimorbidity is necessary and should be intensified in the years to come, which may contribute to understanding a common underlying mechanism of the many facets of the disease and more efforts to promising disease-modifying therapeutic interventions. Furthermore, there are few formal tools for assessing multimorbidity. Some promising indices of multimorbidity may predict various health statuses. It is essential to develop the holistic assessment of physiological status (frailty), complex polypharmacy, and patient priorities. In addition, interventions and managements targeting multimorbidity need to be focused, yet generic. The appropriate intensity of each component is important. Incorporating interdisciplinary care into clinical practice and lessening fragmentation of care make sense. Efforts to improve functional and social frailty should be the driving aim of neurological disease management due to its prominent adverse impact on health status. However, several linking mechanisms co-existing with cardiovascular disease, metabolic and endocrine disease may also worsen various symptoms, thus intervention must also allow sufficient flexibility to accommodate the idiosyncrasies of each multimorbid older adult. It is also essential to facilitate research on the generalizability of evidence on the principle, pathway, key points, and multi-professional team in intervention practices for older adults with multimorbidity patterns in different settings.

### Limitations

Potential limitations of this study include the name of the cluster, which only reflects the dominant disease in the cluster and should not be interpreted as completely discrete groups. Due to the heterogeneous component of multimorbidity patterns generated in different age groups, we were unable to conduct a direct comparison to determine the statistical difference in health status between younger-old and oldest-old if they suffered from the same multimorbidity pattern. It may hinder the exploration of the effect of aging in multimorbidity. Moreover, our choices of samples and measurements are important to consider the regarding representativeness and comprehensiveness. The older adults in our study were selected from high-end long-term care facilities. Most of them received bachelor’s and above education, accounting for nearly 50%, which may impact the interpretation of results on the association between education level and multimorbidity. Different levels of long-term care facilities should be considered in the process of sampling to address selective bias. In terms of measurements of outcomes, we merely assessed health status. Other core outcomes consisting of health-related quality of life, treatment burden, and frailty are also essential for future assessment and intervention on multimorbidity. As well, the detailed types of disease of each system should also be outlined in future studies for accurate assessment of their relationships within the multimorbidity patterns.

## Conclusion

Our findings revealed that the network of multimorbidity for older adults in long-term care facilities was dominated by cardiovascular disease, metabolic and endocrine disease, neurological disease, and orthopedic disease coexisting with each other. Neurological disease and metabolic and endocrine disease were more prevalent in the younger-old, while orthopedic disease was more commonly seen in the oldest-old. CD-MED was common in both younger-old and oldest-old groups. The female showed a higher association with OD-CD-MED-ND in the younger-old group or OD-CD-MED in the oldest-old group compared with the CD-MED. The potential impact of multimorbidity patterns when containing neurological disease on the overall health status (physical structure, activity, and social participation) was considerably deepened in younger-old and more severe in oldest-old. To gain high-quality primary care for older adults in long-term care facilities, identification of common multimorbidity patterns, their deviations between younger-old and oldest-old, risk factors, and modifiable health status may enlighten prevention efforts involving personalized assessment and developing incorporated interdisciplinary management plans. Interventions targeting multimorbidity need to be focused, yet generic. Efforts to preserve functional and social function should be the driving aim of neurological disease management due to its prominent adverse impact on health status. Future research on mechanisms underpinning the development of multimorbidity, holistic assessment of physiological status (frailty), complex polypharmacy, and patient priorities are essential and should be intensified.

### Supplementary Information

**Additional file 1. **Ratio of distance measure and *BIC* value of different clusters.

## Data Availability

The datasets used or analyzed during the current study are available from the corresponding author on reasonable request.

## Abbreviations

ICF: The international classification of functioning, disability, and health
WHO: World health organization
MMSE: Mini-mental state examination
BBS: Berg balance scale
SPCS: Sensory perception and communication scale
BI: Barthel index
SPS: Social participation scale
ADL: Activities of daily living
BIC: Bayesian information criteria
ANOVA: One-way analysis of variance
LSD: Least-significant difference
CD: Cardiovascular disease
RD: Respiratory disease
MED: Metabolic and endocrine disease
DSD: Digestive system disease
OD: Orthopedic disease
ND: Neurological disease
UD: Urogenital disease
AD: Alzheimer’s disease
