# Supplementary material for: Multimorbidity patterns and the association with health status of the oldest-old in long-term care facilities in China: a two-step analysis
Source: BMC Geriatr. 2023 Dec 13;23:851. doi: 10.1186/s12877-023-04507-8 (PMC10720091; doi:10.1186/s12877-023-04507-8)
Supplement: Supplementary file 1 — Additional file 1. Ratio of distance measure and BIC value of different clusters. [file 12877_2023_4507_MOESM1_ESM.docx]

**Additional file 1. Ratio of distance measure and BIC value of different clusters**

| **Age Group** | **Number of Clusters** | **BIC** | **BIC Change** | **Ratio of BIC Changes** | **Ratio of Distance Measures** |
| --- | --- | --- | --- | --- | --- |
| Younger-old | 1 | 383.965 |  |  |  |
|  | 2 | 291.883 | -92.082 | 1.000 | 1.728 |
|  | 3 | 255.971 | -35.912 | 0.390 | 1.075 |
|  | 4 | **225.456** | -30.515 | 0.331 | **1.921** |
|  | 5 | 229.358 | 3.902 | -0.042 | 1.622 |
|  | 6 | 247.588 | 18.230 | -0.198 | . |
| Oldest-old | 1 | 490.307 |  |  |  |
|  | 2 | 393.550 | -96.757 | 1.000 | 1.539 |
|  | 3 | 344.926 | -48.624 | 0.503 | 1.037 |
|  | 4 | **299.518** | -45.408 | 0.469 | **2.538** |
|  | 5 | 306.224 | 6.706 | -0.069 | 1.023 |
|  | 6 | 313.704 | 7.480 | -0.077 | 1.324 |
|  | 7 | 329.293 | 15.590 | -0.161 | 1.086 |
|  | 8 | 346.856 | 17.563 | -0.182 | 1.044 |
|  | 9 | 365.380 | 18.523 | -0.191 | . |

Note: BIC = Schwarz's Bayesian Criterion.
